# Patient and disease characteristics of type-2 diabetes patients with or without chronic kidney disease: an analysis of the German DPV and DIVE databases

**DOI:** 10.1186/s12933-019-0837-x

**Published:** 2019-03-16

**Authors:** Peter Bramlage, Stefanie Lanzinger, Gesine van Mark, Eva Hess, Simon Fahrner, Christoph H. J. Heyer, Mathias Friebe, Jochen Seufert, Thomas Danne, Reinhard W. Holl

**Affiliations:** 1 Institute for Pharmacology and Preventive Medicine, Bahnhofstrasse 20, 49661 Cloppenburg, Germany; 2 Institut für Epidemiologie und medizinische Biometrie, ZIBMT; Universität Ulm, Ulm, Germany; 3 Deutsches Zentrum für Diabetesforschung e.V, Neuherberg, Munich, Germany; 4 Diabetologische Schwerpunktpraxis Dres. Hess, Worms, Germany; 5 Medizinische Klinik, SRH Klinik Sigmaringen, Pfullendorf, Germany; 6 Diabetespraxis Viersen, Viersen, Germany; 7 Evangelisches Krankenhaus, Oberhausen, Germany; 8 Universitätsklinikum Freiburg, Medizinische Fakultät, Freiburg, Germany; 9 Kinderkrankenhaus auf der Bult, Diabeteszentrum für Kinder und Jugendliche, Hannover, Germany

**Keywords:** Type-2-diabetes, Chronic kidney disease, DPV, DIVE, Patient characteristics, Disease characteristics

## Abstract

**Background:**

To evaluate the characteristics of type 2 diabetes (T2DM) patients with or without chronic kidney disease (CKD) in Germany.

**Methods:**

Using combined DPV/DIVE registry data, the analysis included patients with T2DM at least ≥ 18 years old who had an estimated glomerular filtration rate (eGFR) value available. CKD was defined as an eGFR < 60 mL/min/1.73 m^2^ or eGFR ≥ 60 mL/min/1.73 m^2^ and albuminuria (≥ 30 mg/g). Median values of the most recent treatment year per patient are reported.

**Results:**

Among 343,675 patients with T2DM 171,930 had CKD. Patients with CKD had a median eGFR of 48.9 mL/min/1.73 m^2^ and 51.2% had a urinary albumin level ≥ 30 mg/g. They were older, had a longer diabetes duration and a higher proportion was females compared to patients without CKD (all p < 0.001). More than half of CKD patients (53.5%) were receiving long-acting insulin-based therapy versus around 39.1% of those without (p < 0.001). CKD patients also had a higher rate of hypertension (79.4% vs 72.0%; p < 0.001). The most common antihypertensive drugs among CKD patients were renin-angiotensin-aldosteron system inhibitors (angiotensin converting enzyme inhibitors 33.8%, angiotensin receptor blockers 14.2%) and diuretics (40.2%). CKD patients had a higher rate of dyslipidemia (88.4% vs 86.3%) with higher triglyceride levels (157.9 vs 151.0 mg/dL) and lower HDL-C levels (men: 40.0 vs 42.0 mg/dL; women: 46.4 vs 50.0 mg/dL) (all p < 0.001) and a higher rate of hyperkalemia (> 5.5 mmol/L: 3.7% vs. 1.0%). Comorbidities were more common among CKD patients (p < 0.001).

**Conclusion:**

The results illustrate the prevalence and morbidity burden associated with diabetic kidney disease in patients with T2DM in Germany. The data call for more attention to the presence of chronic kidney disease in patients with diabetes, should trigger intensified risk factor control up and beyond the control of blood glucose and HbA1c in these patients. They may also serve as a trigger for future investigations into this patient population asking for new treatment options to be developed.

**Electronic supplementary material:**

The online version of this article (10.1186/s12933-019-0837-x) contains supplementary material, which is available to authorized users.

## Background

The prevalence of chronic kidney disease (CKD) has increased in recent decades alongside an increase in diabetes and hypertension, the main drivers of CKD [1]. Kidney disease attributable to diabetes mellitus (diabetic kidney disease; DKD) is one of the most common complications of diabetes and affects approximately 40% of patients with type 2 diabetes (T2DM) [2, 3]. It can ultimately lead to end-stage renal disease and is associated with an increased risk of cardiovascular disease and death [4–6]. Moreover, people with diabetes can also develop CKD due to etiologies other than diabetes and some may have a combination of DKD and non-diabetic CKD [7]. The prevalence of T2DM is increasing worldwide [8, 9] and consequently diabetes-associated CKD is a major contributor to the global burden of disease [4].

The prevalence of diabetes and CKD and associated healthcare costs vary between different regions of the world [2, 8, 10], and it is therefore important to understand the epidemiology of diabetes-associated CKD and patient characteristics within specific regions and/or countries. In Germany it is estimated that up to 10% of people have been diagnosed with T2DM [11–14] and approximately 40% of individuals with T2DM have comorbid CKD [15].

The aim of the current study was to evaluate the epidemiology of T2DM-associated CKD in Germany and compare the characteristics of patients with or without CKD, using data from the *Diabetes*-*Patienten*-*Verlaufsdokumentation* (DPV) and *DIabetes Versorgungs*-*Evaluation* (DIVE) registries.

## Methods

### Study design and data sources

This analysis used combined data from the DPV and DIVE registries [16–19]. Their design has been described previously. In short, the DPV initiative collects data on patients with diabetes mellitus from centers predominantly in Germany and Austria [18–20]. Data are collected every 6 months using DPV software and the anonymized data are sent to the University of Ulm for aggregation into the database. The DPV initiative, which was established in 1995, was approved by the ethics committee of the University of Ulm, and data collection was approved by local review boards.

The DIVE registry was established in Germany in 2011 [16, 17, 21]. Consecutive patients with diabetes mellitus, regardless of their disease stage, were enrolled from centers across the country, and continue to be followed up. Data are entered into an online database using DIAMAX (Axaris, Ulm, Germany) or DPV software. The protocol was approved by the ethics committee of the Medical School of Hannover, and all patients included in the DIVE registry provided written informed consent.

A total of 394 centers were included in the present analysis (382 Germany, 11 Austria, 1 Luxemburg). Patients were sampled in March 2018 (DPV) and May 2018 (DIVE). and included in the current analysis if they had type-2 diabetes mellitus (T2DM), were at least 18 years old, registered between 2000 and 2017 and had an estimated glomerular filtration rate (eGFR) value calculated according to the modification of diet in renal disease formula (MDRD) available.

### Documentation

For the current analysis, data regarding age, gender, body mass index (BMI), blood pressure, dyslipidemia, type of healthcare provider (office-based/hospital-based), renal parameters, antidiabetic and antihypertensive drug treatment and current comorbidities were collected. For each patient data of the most recent treatment year in the period 2000–2017 was aggregated (median 2013) and analyzed. CKD was defined as eGFR < 60 mL/min/1.73 m^2^ or eGFR ≥ 60 mL/min/1.73 m^2^ and albuminuria (≥ 30 mg/g) [22, 23]. Hypertension was defined as blood pressure (BP) levels above 140 mmHg systolic (SBP) or 90 mmHg diastolic (DBP) or receiving antihypertensive drugs. Dyslipidemia was defined as total cholesterol ≥ 200 mg/dL and/or LDL-C ≥ 160 mg/dL and/or HDL-C < 40 mg/dL and/or triglycerides ≥ 150 mg/dL or receiving lipid-lowering drugs. Coronary artery disease was defined as prior myocardial infarction or angina pectoris.

eGFR was calculated according to the MDRD formula: 175 × creatinine [mg/dL] − 1.154 × age [years] − 0.203 × 0.742 [if female] [24].

### Statistics

Data from all patients were combined and analyzed as a single data set. Categorical variables are presented as percentages. Continuous variables are presented as medians with first and third quartiles (Q1, Q3). T2DM patients with CKD were compared to T2DM patients without CKD. Unadjusted comparisons were conducted using a Chi squared or Kruskal–Wallis test. The false discovery rate method was used to correct p-values for multiple testing. A p-value < 0.05 was considered statistically significant. We also conducted analyses stratified by comorbidity. Statistical analysis was performed using SAS version 9.4.

## Results

**Fig. 1:**
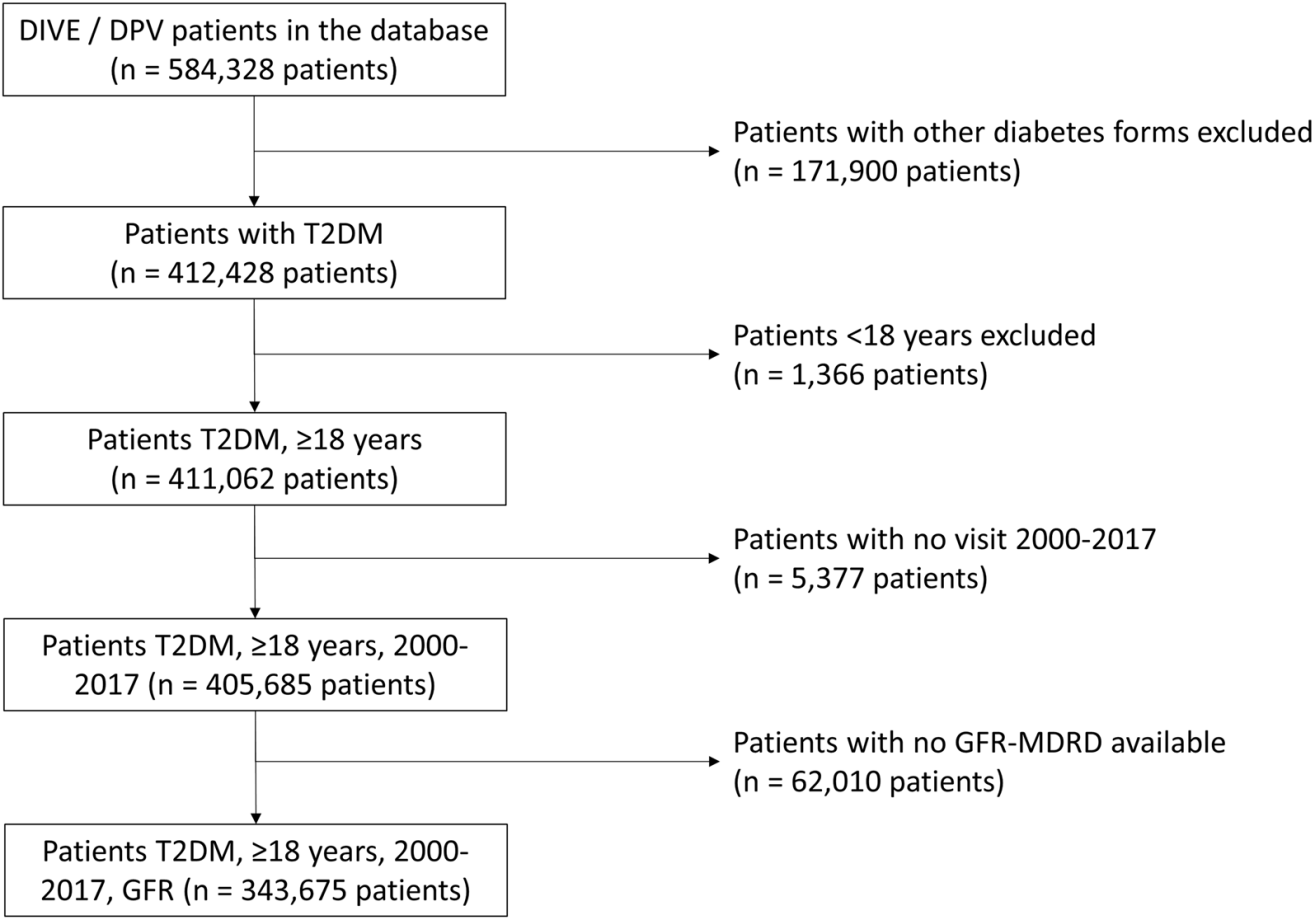
Patient flow chart. *GFR* glomerular filtration rate, *MDRD* modification of diet in renal disease formula, *T2DM* type 2 diabetes mellitus

**Fig. 2:**
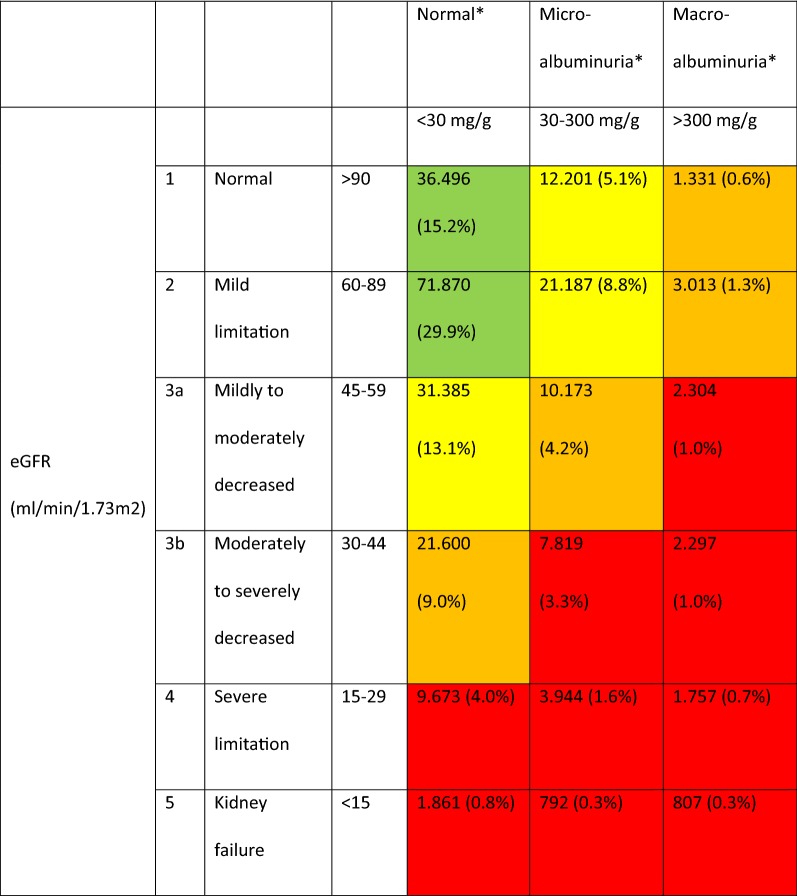
Prevalence of chronic kidney disease by GFR and albuminuria (based on [22]). Green, low risk (if no other markers of kidney disease, no CKD); yellow, moderately increased risk; orange, high risk; red, very high risk. *240.510 patients with information on eGFR category and albuminuria, data are presented as absolute numbers (percent of 240.510). n = 103.165 with missing values on microalbuminuria and/or macroalbuminuria

The analysis population comprised 343,675 patients with T2DM, aged ≥ 18 years, for whom data to compute the GFR-MDRD value were available (Fig. 1), of whom 171,930 had CKD and 171,745 did not have CKD. A total of 108,366 patients were classified as being at low risk, 64,773 patients at moderate risk, 36,117 patients at high and 31,254 patients at very high risk (Fig. 2).

### General characteristics

Patient characteristics are summarized in Table 1 for the overall study population and for patients with or without CKD. T2DM patients with CKD were more likely than those without CKD to be treated by a hospital-based physician (68.5% vs 59.7%, p < 0.001), were older than those without CKD (median 74.5 vs 65.5 years, p < 0.001), had a longer median duration of diabetes (10.3 vs 7.2 years, p < 0.001), and were more likely to be female (52.4% vs 42.0%, p < 0.001).

**Table 1:** Patient characteristics (overall study population and according to presence/absence of CKD)

	n	Total T2DM	T2DM + CKD^b^	T2DM − CKD^b^	p-value
		(n = 343,675)	(n = 171,930)	(n = 171,745)	
Healthcare provider					
Office based in %	343,675	35.9	31.5	40.3	< 0.001
Hospital based in %	343,675	64.1	68.5	59.7	< 0.001
Age in years	343,675	70.3 (60.4; 78.0)	74.5 (66.2;80.7)	65.5 (56.2; 74.1)	< 0.001
Female gender in %	343,675	47.2	52.4	42.0	< 0.001
Weight in kg	321,929	85.5 (74.0; 100.0)	84.7 (73.0; 98.3)	87.0 (75.0; 101.0)	< 0.001
Body mass index in kg/m^2^	314,804	29.9 (26.3; 34.4)	29.9 (26.3; 34.4)	29.9 (26.4; 34.4)	0.027
Blood pressure					
Systolic BP in mmHg	330,285	132.5 (121.0; 145.0)	132.5 (120.0; 145.0)	132.5 (122.0; 144.0)	0.007
Systolic BP ≥ 140 mmHg in %	330,285	41.5	41.7	41.4	< 0.001
Diastolic BP in mmHg	329,769	80.0 (70.0; 81.0)	78.0 (70.0; 80.0)	80.0 (70.0; 83.0)	< 0.001
Diastolic BP ≥ 90 mmHg in %	329,769	14.0	12.2	15.9	< 0.001
AntiHT drug treatment in %	343,675	56.7	62.6	20.7	< 0.001
Hypertension^a^ in %	335,762	75.7	79.4	72.0	< 0.001
Dyslipidemia	285,009	87.4	88.4	86.3	< 0.001
LDL-C in mg/dL	236,106	108.3 (83.0, 137.0)	105.0 (79.3; 135.0)	112.1 (86.8; 139.2)	< 0.001
TC in mg/dL	263,462	185.6 (154.7; 218.5)	181.8 (150.8; 216.0)	189.0 (159.0; 220.4)	< 0.001
TG in mg/dL	254,288	155.0 (110.0; 223.7)	157.9 (113.0; 228.1)	151.0 (107.0; 219.3)	< 0.001
HDL-C in men in mg/dL	128,099	41.0 (34.0; 50.0)	40.0 (33.0; 48.5)	42.0 (34.8; 50.3)	< 0.001
HDL-C in women in mg/dL	110,834	46.4 (37.5; 58.0)	46.4 (37.5; 57.0)	50.0 (41.0; 60.0)	< 0.001
Diabetes					
Diabetes duration in years	343,675	9.0 (3.5, 15.4)	10.3 (4.9; 17.2)	7.2 (2.4; 13.4)	< 0.001
0–5 years in %	343,675	32.3	25.5	39.1	< 0.001
6–10 years in %	343,675	22.7	22.2	23.2	< 0.001
> 10 years in %	343,675	45.0	52.3	37.8	< 0.001
HbA1c in %	325,058	7.1 (6.3; 8.4)	7.2 (6.3; 8.3)	7.1 (6.3; 8.4)	< 0.001
HbA1c < 6.5%	325,058	31.6	30.5	32.7	< 0.001
HbA1c < 7.0%	325,058	46.5	45.8	47.3	< 0.001
Kidney parameters					
Potassium					
≤ 4.8 mmol/L in %	73,615	85.4	81.8	89.8	< 0.001
> 4.8– ≤ 5.5 mmol/L in %	73,615	12.2	14.6	9.3	< 0.001
> 5.5– ≤ 6.0 mmol/L in %	73,615	1.6	2.4	0.7	< 0.001
> 6.0 mmol/L in %	73,615	0.8	1.3	0.3	< 0.001
Urinary albumin in mg/g					
Normal (< 30 mg/g) in %	240,510	71.9	48.8	100.0	< 0.0001
Micro (≥ 30–300 mg/g) in %	240,510	22.5	40.8	0.0	< 0.001
Macro (> 300 mg/g) in %	204,211	5.6	10.4	0.0	< 0.001
Creatinine in mg/dL	343,513	1.0 (0.8; 1.3)	1.3 (1.0; 1.6)	0.8 (0.7; 1.0)	< 0.001
eGFR mL/min/1.73 m^2^	343,675	67.9 (48.9; 85.9)	48.9 (36.3; 59.0)	81.6 (71.0; 96.5)	< 0.001
< 15	343,675	1.5	3.0	0.0	< 0.001
15 to < 30 in %	343,675	6.3	12.6	0.0	< 0.001
30 to < 45 in %	343,675	13.0	25.9	0.0	< 0.001
45 to < 60 in %	343,675	18.3	36.6	0.0	< 0.001
60 to < 89 in %	343,675	40.2	14.1	66.3	< 0.001
≥ 90 in %	343,675	20.8	7.9	33.7	< 0.001

Median (Q1; Q3) or percent (%)

*AntiHT* antihypertensive, *BP* blood pressure, *CKD* chronic kidney disease, *eGFR* estimated glomerular filtration rate, *HDL-C* high-density lipoprotein cholesterol, *LDL-C* low-density lipoprotein cholesterol, *TC* total cholesterol, *TG* triglycerides, *T2DM* type 2 diabetes mellitus

^a^Defined as either systolic BP ≥ 140 mmHg OR diastolic BP ≥ 90 mmHg OR on antihypertensive drug treatment

^B^defined as eGFR < 60 mL/min/1.73 m^2^ OR eGFR ≥ 60 mL/min/1.73 m^2^ and albuminuria (≥ 30 mg/g)

Patients with CKD had a higher rate of hypertension (79.4% vs 72.0%, p < 0.001), they were more likely to be receiving antihypertensive drugs (62.6% vs 20.7%, p < 0.001) and their median BP value was (slightly) lower than those for patients without CKD (Table 1). Both groups had evidence of high levels of diabetic dyslipidemia, with elevated triglyceride levels and low HDL-C levels; median values were significantly worse in patients with CKD than in those without CKD (triglycerides: 157.9 vs 151.0 mg/dL; HDL-C in men: 40.0 vs 42.0 mg/dL; HDL-C in women: 46.4 vs 50.0 mg/dL; both p < 0.001). As would be expected, patients with CKD had significantly worse values for parameters reflecting kidney function/damage than those without CKD. The rate of hyperkalemia (> 5.5 mmol/L) was 3.7% versus 1.0% (p < 0.001).

Patient characteristics for the whole study population (i.e. irrespective of CKD status) stratified by region of Germany (north, south, west, east) are summarized in Additional file 1: Table S1.

### Drug treatment

Antidiabetic and antihypertensive drug treatments received by T2DM patients with or without CKD are summarized in Table 2. With respect to antidiabetic treatment, patients with CKD were more likely than those without CKD to be prescribed glinides (3.9% vs 3.0%, p < 0.001) and insulin (short-acting insulin: 51.4% vs 36.7%; long-acting insulin 53.5% vs 39.1%; both p < 0.001). All other drug classes were more common in those without CKD, most notably metformin (28.6% vs 47.2%). Patients with CKD were less likely than those without CKD to be receiving ≥ 2 antidiabetic drugs (15.0% vs 21.0%, p < 0.001).

**Table 2:** Drug treatment by class (overall study population and according to presence/absence of CKD)

	Total T2DM	T2DM + CKD^a^	T2DM − CKD^a^	p-value
	(n = 343,675)	(n = 171,930)	(n = 171,745)	
Antidiabetic drugs				
Metformin in %	37.9	28.6	47.2	< 0.001
Sulfonylurea in %	11.4	11.2	11.7	< 0.001
Alpha-glucosidase inhibitors in %	1.2	1.3	1.1	< 0.001
DPP-4 inhibitors in %	14.7	14.5	14.9	0.004
Glinides in %	3.5	3.9	3.0	< 0.001
SGLT-2 inhibitors in %	2.6	2.0	3.2	< 0.001
GLP-1 RA in %	3.0	2.4	3.6	< 0.001
Glitazones in %	1.1	1.0	1.2	< 0.001
Short-acting insulin in %	44.0	51.4	36.7	< 0.001
Long-acting insulin in %	46.3	53.5	39.1	< 0.001
≥ 2 antidiabetic drugs in %	18.0	15.0	21.0	< 0.001
Antihypertensive drugs				
Angiotensin converting enzyme inhibitors in %	31.2	33.8	28.6	< 0.001
Angiotensin receptor blockers in %	12.3	14.2	10.3	< 0.001
Beta-blockers in %	31.3	36.7	25.8	< 0.001
Calcium channel blockers in %	16.2	19.5	12.9	< 0.001
Diuretics in %	31.6	40.2	23.1	< 0.001
≥ 2 antihypertensive drugs in %	39.9	47.3	32.5	< 0.001

Percent (%)

*CKD* chronic kidney disease, *DPP-4* dipeptidyl peptidase-4, *GLP-1 RA* glucagon-like peptide-1 receptor agonist, *SGLT-2* sodium–glucose co-transporter-2, *T2DM* type 2 diabetes mellitus

^a^Defined as eGFR < 60 mL/min/1.73 m^2^ OR eGFR ≥ 60 mL/min/1.73 m^2^ and albuminuria (≥ 30 mg/g)

Consistent with the higher rate of hypertension seen among T2DM patients with CKD, patients with CKD were more likely than those without CKD to be receiving antihypertensive drugs (p < 0.001 for all classes) and to be receiving ≥ 2 drugs (49.2% vs 33.6%, p < 0.001). The most common antihypertensive drugs prescribed to patients with CKD were renin–angiotensin–aldosterone system (RAAS) blockers (comprising angiotensin converting enzyme [ACE] inhibitors 33.8% and angiotensin receptor blockers [ARBs] 14.2%), followed by diuretics (40.2%) and beta-blockers (36.7%). The most common drugs among patients without CKD were also RAAS blockers (comprising ACE inhibitors 28.6% and ARBs 10.3%), followed by beta-blockers (25.8%) and diuretics (23.1%).

### Comorbidities

The rates of all comorbidities—stroke, retinopathy, coronary artery disease (including myocardial infarction), peripheral artery disease and diabetic foot complications (including amputations)—were significantly higher among T2DM patients with CKD compared to those without CKD (all p < 0.001); Table 3.

**Table 3:** Comorbidity (overall study population and according to presence/absence of CKD)

	n	Total T2DM	T2DM + CKD^a^	T2DM − CKD^a^	p-value
		(n = 343,675)	(n = 171,930)	(n = 171,745)	
Prior stroke in %	343,675	7.6	9.2	6.1	< 0.001
Retinopathy in %	343,675	5.2	6.5	4.0	< 0.001
Proliferative in %	343,675	1.9	2.5	1.3	< 0.001
Coronary artery disease in %	343,675	8.9	10.6	7.3	< 0.001
Prior myocardial infarction in %	343,675	8.3	9.9	6.8	< 0.001
Peripheral artery disease in %	343,675	16.5	20.7	12.3	< 0.001
Diabetic foot complications in %	343,675	11.3	13.4	9.1	< 0.001
Minor amputation in %	343,675	2.1	2.8	1.4	< 0.001
Major amputation in %	343,675	0.9	1.2	0.7	< 0.001

Percent (%)

*CKD* chronic kidney disease, *T2DM* type 2 diabetes mellitus

^a^Defined as eGFR < 60 mL/min/1.73 m^2^ OR eGFR ≥ 60 mL/min/1.73 m^2^ and albuminuria (≥ 30 mg/g)

Patient characteristics stratified by comorbidity for the overall study population are summarized in Table 4. The majority of patients with comorbidities were being treated by hospital-based physicians, with the highest rates seen for patients with prior stroke (74.7%) CAD (69.5%) or CKD (68.5%). The proportion of female patients was highest for patients with CKD (52.4% vs 36.4–46.2% for other comorbidities). Median duration of diabetes was slightly shorter in those with stroke, CAD or CKD (10.3–10.6 years) than in those with peripheral artery disease, foot complications or retinopathy (12.0–16.1 years). The rates of hypertension and antihypertensive drug treatment were slightly lower for patients with diabetic foot complications (hypertension 79.5%; treatment 64.0%) and CKD (hypertension 79.4%; treatment 62.6%) than for patients with other comorbidities (hypertension 82.5–85.3%; treatment 70.0–75.4%). Median triglyceride level was higher in those with CKD compared with those with other comorbidities (157.9 vs 148.9–152.0 mg/dL).

**Table 4:** Patient characteristics by comorbidity (overall study population)

	Prior stroke	Retinopathy	CAD	CKD^b^	Diabetic foot complications	PAD
	(n = 26,270)	(n = 18,036)	(n = 30,748)	(n = 171,930)	(n = 38,765)	(n = 56,741)
Healthcare provider						
Office based in %	25.3	36.9	30.5	31.5	49.5	44.3
Hospital based in %	74.7	63.1	69.5	68.5	50.5	55.7
Age in years	75.1 (67.8; 81.0)	71.1 (63.1; 78.0)	73.2 (65.4; 79.6)	74.5 (66.1; 80.7)	72.8 (64.3; 79.2)	73.9 (65.9; 80.1)
< 65 years	18.1	29.9	23.7	22.4	26.7	22.6
≥ 65 years	81.9	70.1	76.3	77.6	73.3	77.4
Female gender in %	46.0	46.2	36.4	52.4	39.3	42.0
Weight in kg	82.0 (71.9; 94.8)	86.5 (75.0; 100.0)	85.0 (74.3; 98.0)	84.7 (73.0; 98.3)	88.0 (75.4; 103.0)	85.0 (74.0; 99.4)
Body mass index in kg/m^2^	29.0 (25.6; 32.9)	30.5 (26.8; 34.8)	29.4 (26.2; 33.5)	29.9 (26.3; 34.4)	30.1 (26.3; 34.6)	29.7 (26.1; 34.1)
Blood pressure						
Systolic BP in mmHg	135.0 (123.0; 146.5)	135.0 (125.0; 147.5)	130.0 (120.0; 140.0)	132.5 (120.0; 145.0)	134.0 (125.0; 145.0)	134.0 (123.0; 145.0)
Systolic BP ≥ 140 mmHg in %	44.5	45.9	36.1	41.7	41.3	41.2
Diastolic BP in mmHg	77.5 (70.0; 80.0)	79.0 (70.0; 80.0)	75.0 (70.0; 80.0)	78.0 (70.0; 80.0)	77.0 (70.0; 80.0)	75.5 (70.0; 80.0)
Diastolic BP ≥ 90 mmHg in %	12.4	12.2	9.2	12.2	9.9	10.0
AntiHT drug treatment in %	72.5	71.0	75.4	62.6	64.0	70.0
Hypertension^a^ in %	85.3	84.2	85.2	79.4	79.5	82.5
Dyslipidemia in %	90.2	87.5	91.4	88.4	85.4	87.4
LDL-C in mg/dL	104.0 (79.0; 133.0)	104.4 (80.0; 132.0)	96.0 (73.5; 123.7)	105.0 (79.3; 135.0)	104.0 (80.0; 130.0)	104.0 (79.3; 131.5)
TC in mg/dL	177.9 (147.0; 211.5)	182 (152.7; 214.0)	168.0 (140.0; 201.1)	181.7 (150.8; 216.0)	176.0 (147.0; 208.0)	177.9 (149.0; 211.0)
TG in mg/dL	151.0 (109.0; 215.0)	152.0 (109.0; 217.0)	151.0 (109.0; 218.0)	157.9 (113.0; 228.1)	148.9 (105.3; 210.5)	150.6 (108.0; 215.2)
HDL-C in men in mg/dL	40.0 (34.0; 49.0)	42.0 (35.0; 50.3)	39.5 (33.0; 48.0)	40.0 (33.0, 48.5)	41.0 (34.6; 50.3)	41.3 (34.5; 50.3)
HDL-C in women in mg/dL	46.0 (37.0; 56.1)	47.0 (38.7; 58.0)	45.0 (37.0; 55.5)	46.4 (37.5; 57.0)	48.0 (39.0; 58.5)	47.0 (38.7; 58.0)
Diabetes						
Diabetes duration in years	10.5 (5.2; 17.3)	16.1 (9.9; 23.7)	10.6 (5.1; 18.0)	10.3 (4.9; 17.2)	13.2 (7.5; 20.3)	12.0 (6.5; 19.4)
0–5 years in %	24.1	10.6	24.8	25.5	15.9	18.9
6–10 years in %	22.4	14.9	21.5	22.2	19.6	21.4
> 10 years in %	53.4	74.5	53.7	52.3	64.5	59.7
HbA1c in %	7.1 (6.3; 8.2)	7.5 (6.6; 8.6)	7.1 (6.3; 8.2)	7.1 (6.3; 8.3)	7.1 (6.3; 8.1)	7.0 (6.3; 8.1)
HbA1c < 6.5% in %	31.7	21.7	32.2	30.5	30.7	32.5
HbA1c < 7.0% in %	47.6	36.7	48.0	45.8	47.6	48.9
Kidney parameters						
Potassium in mmol/L	4.3 (3.9; 4.6)	4.4 (4.0; 4.7)	4.3 (4.0; 4.7)	4.3 (3.9; 4.7)	4.4 (4.1; 4.8)	4.3(4.0; 4.7)
≤ 4.8 mmol/L in %	85.4	81.9	83.2	81.8	79.9	81.6
> 4.8–≤ 5.5 mmol/L in %	11.9	15.6	14.1	14.6	16.9	15.1
> 5.5– ≤ 6.0 mmol/L in %	1.7	1.7	1.8	2.3	2.3	2.2
> 6.0 mmol/L in %	1.1	0.7	0.9	1.3	0.9	1.0
Urinary albumin in mg/g						
Normal (< 30 mg/g) in %	70.0	66.7	71.1	48.8	66.3	65.9
Micro (≥ 30–300 mg/g) in %	23.1	24.9	22.5	40.8	26.4	26.5
Macro (> 300 mg/g) in %	6.9	8.4	6.4	10.4	7.3	7.6
Creatinine in mg/dL	1.1 (0.9; 1.4)	1.0 (0.8; 1.4)	1.1 (0.9; 1.4)	1.3 (1.0; 1.6)	1.1 (0.9 1.4)	1.1 (0.9; 1.5)
eGFR mL/min/1.73 m^2^	59.4 (42.4; 76.8)	60.9 (42.8; 79.8)	59.6 (42.5; 76.0)	48.9 (36.3; 59.0)	61.5 (43.1; 81.1)	59.2 (41.7; 77.7)
< 15	2.0	2.1	1.7	3.0	2.2	2.2
15 to < 30 in %	8.8	9.3	9.0	12.6	8.5	9.4
30 to < 45 in %	17.8	16.6	17.6	25.9	16.7	17.9
45 to < 60 in %	22.5	20.7	22.3	36.6	20.4	21.7
60 to < 90 in %	35.9	35.7	37.3	14.1	36.1	35.4
≥ 90 in %	13.1	15.7	12.1	7.9	16.2	13.4

Median (Q1; Q3) or percent (%)

Patients with multiple comorbidities possible

*AntiHT* antihypertensive, *BP* blood pressure, *CAD* coronary artery disease, *CKD* chronic kidney disease, *eGFR* estimated glomerular filtration rate, *HDL-C* high-density lipoprotein cholesterol, *LDL-C* low-density lipoprotein cholesterol, *PAD* peripheral artery disease, *TC* total cholesterol, *TG* triglycerides

^a^Defined as either systolic BP ≥ 140 mmHg OR diastolic BP ≥ 90 mmHg OR on antihypertensive drug treatment

^b^Defined as eGFR < 60 mL/min/1.73 m^2^ OR eGFR ≥ 60 mL/min/1.73 m^2^ and albuminuria (≥ 30 mg/g)

Drug treatment stratified by comorbidity for the overall study population is summarized in Additional file 2: Table S2. The most noticeable differences between patients with CKD and those with other comorbidities were a lower rate of metformin use (28.6% vs 30.8–32.5%) and lower rate of use of ≥ 2 antidiabetic drugs in those with CKD or retinopathy (15.0% vs 15.6–17.6%).

## Discussion

Diabetes is the leading cause of CKD worldwide, and despite the use of current antidiabetic and antihypertensive therapies, the risk remains high [4]. The presence of CKD makes a substantial contribution to the socioeconomic burden associated with diabetes [25, 26].

Estimates of the prevalence of T2DM in Germany range from 5 to 10% depending on the diagnostic criteria used [11, 12, 14, 27] and it is projected to increase to 16% by 2040 among people aged ≥ 40 years [14]. According to the German Health Interview and Examination Survey for Adults 2008–2011 (DEGS1), the prevalence of comorbid CKD among adults with T2DM in Germany is approximately 40% [15], which is in line with global estimates [4]. DEGS1 also showed that 2.3% of the adult population of Germany (i.e. more than 2 million people) has at least moderate impairment of renal function (eGFR < 60 mL/min/1.73 m^2^), and the prevalence was 2.25-fold higher among people with diabetes compared with those without diabetes [28]. Analysis of the German Chronic Kidney Disease (GCKD) cohort indicated that 35% of patients with moderate CKD who are under specialist care in Germany have diabetes, and that diabetic nephropathy is considered the leading cause of kidney disease in 41% of that subgroup of patients [29]. People with diabetes can develop CKD not only as a consequence of their diabetes, but also due to other etiologies, and can have a combination of diabetic kidney disease and non-diabetic CKD [7].

The current study analyzed DPV/DIVE data for 343,675 adults with T2DM who had a GFR-MDRD value available and found a prevalence of CKD of 50.0%, which is higher compared with previous estimates [15]. Among T2DM patients with CKD included in the study, median eGFR was 48.9 mL/min/1.73 m^2^, 51.2% had micro or macroalbuminuria and 3.7% had hyperkalemia (> 5.5 mmol/L) vs 1% in the T2DM population without CKD. As reflected in Fig. 2 of the present paper, the majority of patients (n = 94,412) had their diagnosis being made based on an eGFR < 60 mg/min/1.73 m^2^ only. Further 37,732 had albuminuria ≥ 30 mg/g while having an eGFR ≥ 60 mg/min/1.73 m^2^, and 33,388 patients had their diagnosis made based on the presence of microalbuminuria alone. Further markers for the identification of patients with chronic kidney disease in the presence of diabetes as well as the identification of those with diabetic kidney disease would be of interest, such as plasma copeptin [30] and prognostics markers such as symmetric and asymmetric dimethylarginine [31], but these were not contained in the present dataset.

The study compared the characteristics of T2DM patients with and without CKD. T2DM patients with CKD were significantly older, had a longer duration of diabetes and were more likely to be female than those without CKD. Older age is a recognized risk factor for CKD [2, 4]. CKD is generally considered to be more common among men than women [2, 4], although the United Kingdom Prospective Diabetes Study (UKPDS) identified female sex as a risk factor for impaired renal function [32]. While weight was slightly higher among patients with CKD in our study, there was no association of BMI with the level of CKD as suggested by prior research [33]. The current study also found that in Germany, T2DM patients with CKD were significantly more likely than those without CKD to be under the care of a hospital-based physician.

Based on median HbA1c values, the overall level of glycemic control appeared to be generally acceptable, and comparable among patients with CKD compared with those without CKD (median 7.2 vs. 7.1%, p < 0.001). This is important as HbA1c trajectories have been associated with renal disease progression [34]. Although a wide range of antidiabetic medications were prescribed to patients in both the CKD and non-CKD groups, it was notable that more than 50% of the patients with CKD were receiving long-acting insulins compared with < 40% of those without CKD. It has been reported elsewhere that only 31% of a general German T2DM population were prescribed insulin-based therapies [13]. The findings of the current study are consistent with an analysis of the GCKD cohort, which also found that while antidiabetic treatment patterns for T2DM patients with CKD varied, more than 50% were receiving insulin-based therapies [35]. Similar to the current study, overall metabolic control appeared satisfactory in the GCKD cohort, with the median HbA1c value being 7.0% [35]; however, it was found that use of insulin was associated with an increased HbA1c value > 7.0% [35].

In patients with T2DM, hypertension increases the risk of albuminuria, impaired renal function, end-stage renal disease and death [32, 36, 37]. A diagnosis of hypertension was common in the T2DM population enrolled in the current study and was significantly more frequent among those with CKD than among those without CKD. However, median BP values were lower in patients with CKD, presumably because they were more likely to be receiving antihypertensive medication. The most common classes of antihypertensive drugs prescribed to T2DM patients with CKD were RAAS blockers (ACE inhibitors 33.8%, angiotensin receptor blockers 14.2%), diuretics (40.2%) and beta-blockers (36.7%).

RAAS blockade is recommended for diabetic patients with hypertension, including those with CKD [38]. Treatment with either an ACE inhibitor or an ARB reduces the progression of CKD in patients with macroalbuminuria; however, combining these two drug classes provides no additional benefit in terms of outcomes and increases the risk of adverse events [39–41]. Adding a mineralocorticoid receptor antagonist (MRA) to an RAAS blocker reduces proteinuria further in patients with CKD [42, 43], but steroidal MRAs are associated with adverse effects, including an increased risk of hyperkalemia [44]. There is no information about the use of MRA in this analysis, as MRA are documented as diuretics only without further specification.

Dyslipidemia affects at least 75% of patients with T2DM [45] and lipid levels are generally worse in T2DM patients with CKD compared with those without CKD [46]. The results of the current study are consistent with this: more than 85% of patients in both groups had dyslipidemia, but median triglyceride and HDL-C levels were significantly worse in those with CKD than those without CKD.

Diabetes is associated with both microvascular complications (nephropathy, retinopathy and neuropathy) and macrovascular complications (including atherosclerotic disorders and impaired cardiac function) [47]. Both diabetes and CKD are associated with an increased risk of cardiovascular disease, and the risk is particularly high in patients who have CKD [48]. CKD also increases the risk of mortality compared with T2DM patients without kidney disease [48, 49]. In the current study, comorbidities, including stroke, coronary artery disease and peripheral artery disease, as well as retinopathy and diabetic foot complications were significantly more common in T2DM patients with CKD compared to those without CKD.

Statistical comparison of the characteristics of patients with different comorbidities was not undertaken, but when stratified by comorbidity, there was a greater proportion of female patients and a higher median triglyceride level in the CKD subgroup compared with other comorbidity subgroups. Patients with CKD or prior stroke were most likely to be treated by a hospital-based physician. Median duration of diabetes was slightly shorter in patients with CKD, stroke or CAD compared with those with peripheral artery disease, foot complications or retinopathy. Hypertension and antihypertensive treatment appeared to be less common among patients with CKD or diabetic foot complications compared with those with other comorbidities. The rates of metformin use and use of ≥ 2 antidiabetic drugs were lower in the CKD subgroup compared with other comorbidity subgroups, while the rate of antihypertensive drug use was lower among patients with CKD or diabetic foot complications compared with those with other comorbidities.

Regional differences in the prevalence and characteristics of patients with T2DM have been noted in Germany, which are thought to relate to differences in the distribution of risk factors, regional deprivation and individual socioeconomic status [12, 27, 50]. The current study included a comparison of the characteristics of the overall T2DM study population (irrespective of their CKD status) between different regions of Germany. Statistical comparisons were not performed, but some potentially interesting differences were noted. Although patients in the eastern region were most likely to be treated by a hospital-based physician, this region had the lowest rate of attainment of HbA1c < 7.0% (closely followed by the north). T2DM patients in the eastern region also had the lowest median eGFR, highest rate of albuminuria > 300 mg/g and highest rate of hyperkalemia, as well as the highest median triglyceride and lowest median HDL-C levels. The rate of hypertension was highest in the northern region. Such information may be useful for healthcare planning within different areas of the country.

The main limitation of this study is that patients were recruited from specialized centers that were participating in diabetes registries, which could bias the results towards patients requiring specialist care. In addition, the cross-sectional nature of the study precludes the identification of causal links between findings. We also were not able to establish a causal relationship between diabetes and CKD which would enable the identification of a cohort of patients with diabetic kidney disease. Finally, we did not verify the diagnosis of albuminuria on a subsequent occasion (or checked whether negative tests would have been positive), leaving room for variation of the true prevalence of patients with an eGFR ≥ 60 mL/min/1.73 m^2^ and albuminuria 30–300 mg/g. Strengths include the large number of participants and the routine clinical practice setting which means that the study provides evidence from real-world care. No data on the ethnicity of patients were recorded [51].

## Conclusions

In conclusion, this study describes the prevalence and associated morbidity burden associated with diabetic kidney disease in Germany. The data call for more attention to the presence of chronic kidney disease in patients with diabetes, should trigger intensified risk factor control up and beyond the control of blood glucose and HbA1c in these patients. They may also serve as a trigger for future investigations into this patient population asking for new treatment options to be developed.

## Additional files

**Additional file 1: Table S1.** Patient characteristics by region of Germany (overall study population). Legend: Median (Q1; Q3) or percent (%). BP = blood pressure; eGFR = estimated glomerular filtration rate; HDL-C = high-density lipoprotein cholesterol; LDL-C = low-density lipoprotein cholesterol; TC = total cholesterol; TG = triglycerides; T2DM = type 2 diabetes mellitus.

**Additional file 2: Table S2.** Drug treatment by comorbidity. Legend: Percent (%). ^‡^Defined as eGFR < 60 ml/min/1.73 m^2^ OR eGFR ≥ 60 ml/min/1.73 m^2^ and overt albuminuria. CKD = chronic kidney disease; DPP-4 = dipeptidyl peptidase-4; GLP-1 RA = glucagon-like peptide-1 receptor agonist; SGLT-2 = sodium-glucose co-transporter-2.

## Abbreviations

ACE: angiotensin converting enzyme
AntiHT: antihypertensive
BMI: body mass index
BP: blood pressure
CAD: chronic artery disease
CKD: chronic kidney disease
DBP: diastolic blood pressure
DEGS: Deutsche-Erwachsenen-Gesundheitsstudie
DIVE: Diabetes Versorgungs-Evaluation
DPP-4: dipeptidyl peptidase-4
DPV: Diabetes Patienten-Verlaufsdokumentation
GCKD: German chronic kidney disease
GLR-1 RA: Glucagon-like peptide-1 receptor agonist
eGFR: estimated glomerular filtration rate
GFR: glomerular filtration rate
HDL-C: high-density lipoprotein cholesterol
LDL-C: low-density lipoprotein cholesterol
MDRD: modification of diet in renal disease formula
MRA: mineralocorticoid receptor antagonist
PAD: peripheral artery disease
RAAS: renin angiotensin aldosterone system
SBP: systolic blood pressure
SGLT-2: sodium–glucose co transporter-2
T2DM: type 2 diabetes
TC: total cholesterol
TG: triglycerides
UKPDS: United Kingdom Prospective Diabetes Study
