# Supplementary material for: Patient and disease characteristics of type-2 diabetes patients with or without chronic kidney disease: an analysis of the German DPV and DIVE databases
Source: Cardiovasc Diabetol. 2019 Mar 16;18:33. doi: 10.1186/s12933-019-0837-x (PMC6420726; doi:10.1186/s12933-019-0837-x)
Supplement: Supplementary file 1 — Additional file 1: Table S1. Patient characteristics by region of Germany (overall study population). Legend: Median (Q1; Q3) or percent (%). BP = blood pressure; eGFR = estimated glomerular filtration rate; HDL-C = high-density lipoprotein cholesterol; LDL-C = low-density lipoprotein cholesterol; TC = total cholesterol; TG = triglycerides; T2DM = type 2 diabetes mellitus. [file 12933_2019_837_MOESM1_ESM.docx]

**Table S1:** Patient characteristics by region of Germany (overall study population)

|  | North | South | West | East |
| --- | --- | --- | --- | --- |
|  | (n=36,556) | (n=99,418) | (n=157,379) | (n=40,935) |
| Treating physician |  |  |  |  |
| Office based in % | 38.1 | 40.3 | 31.8 | 24.3 |
| Hospital based in % | 61.6 | 59.7 | 68.2 | 75.7 |
| Age in years | 68.2 (58.6; 76.1) | 70.5 (60.3; 78.3) | 71.0 (61.0; 78.6) | 70.4 (60.5; 77.7) |
| Female gender in % | 45.4 | 46.8 | 47.6 | 48.5 |
| Weight in kg | 88.5 (76.1; 103.0) | 85.0 (73.0; 98.5) | 85.0 (74.0; 100.0) | 86.0 (74.5; 100.0) |
| Body mass index in kg/m^2^ | 30.5 (26.8; 35.0) | 29.7 (26.2; 34.1) | 29.8 (26.2; 34.3) | 30.1 (26.6; 34.7) |
| Blood pressure |  |  |  |  |
| Systolic BP in mmHg | 134.0 (122.5; 145.0) | 133.0 (122.0; 145.0) | 130.0 (120.0; 142.0) | 135.0 (125.0; 148.0) |
| Systolic BP ≥140 mmHg in % | 43.0 | 41.7 | 39.6 | 46.5 |
| Diastolic BP in mmHg | 80.0 (70.0; 83.0) | 80.0 (70.0; 82.0) | 80.0 (70.0; 80.0) | 80.0 (70.0; 84.0) |
| Diastolic BP ≥90 mmHg in % | 15.7 | 14.6 | 12.3 | 17.4 |
| Hypertension* in % | 81.2 | 78.0 | 73.1 | 75.7 |
| Dyslipidemia in % | 89.7 | 87.5 | 86.3 | 89.8 |
| LDL-C in mg/dL | 109.4 (84.7; 138.8) | 108.5 (82.0; 137.0) | 109.0 (83.1; 138.0) | 104.4 (79.0; 135.0) |
| TC in mg/dL | 185.6 (155.0; 220.0) | 187.0 (155.0; 219.0) | 184.0 (154.0; 218.0) | 185.6 (154.7; 220.4) |
| TG in mg/dL | 157.9 (112.0; 228.1) | 155.0 (108.0; 225.0) | 153.5 (110.0; 219.3) | 162.0 (114.0; 239.2) |
| HDL-C in men in mg/dL | 41.0 (34.8; 50.0) | 41.0 (34.0; 50.0) | 41.0 (34.0; 50.0) | 38.7 (31.7; 48.3) |
| HDL-C in women in mg/dL | 48.3 (39.1; 58.0) | 48.0 (39.0; 58.0) | 47.0 (38.7; 58.0) | 46.3 (36.7; 56.1) |
| Diabetes |  |  |  |  |
| Diabetes duration in years | 8.4 (3.0; 15.5) | 8.4 (3.1; 14.5) | 9.2 (3.7; 15.6) | 9.7 (3.7; 16.5) |
| 0-5 years in % | 35.2 | 34.2 | 31.3 | 30.6 |
| 6-10 years in % | 20.9 | 23.7 | 22.7 | 21.8 |
| >10 years in % | 43.8 | 42.2 | 46.0 | 47.6 |
| HbA1c in % | 7.3 (6.5; 8.8) | 7.1 (6.3; 8.4) | 7.0 (6.2; 8.2) | 7.4 (6.5; 8.9) |
| HbA1c <6.5% in % | 27.3 | 31.8 | 34.0 | 26.5 |
| HbA1c <7.0% in % | 41.3 | 47.0 | 49.3 | 39.5 |
| Kidney parameters |  |  |  |  |
| Potassium mmol/L | 4.3 (4.0; 4.6) | 4.2 (3.9; 4.6) | 4.3 (4.0; 4.7) | 4.3 (4.0; 4.7) |
| ≤4.8 mmol/L in % | 85.9 | 87.2 | 84.5 | 83.3 |
| >4.8 - ≤5.5 mmol/L in % | 12.2 | 10.6 | 13.0 | 13.5 |
| >5.5 - ≤6.0 mmol/L in % | 1.2 | 1.4 | 1.7 | 2.0 |
| >6.0 mmol/L in % | 0.6 | 0.7 | 0.9 | 1.2 |
| Urinary albumin in mg/g |  |  |  |  |
| Normal (<30 mg/g) in % | 66.7 | 72.5 | 74.1 | 67.4 |
| Micro (≥30-300 mg/g) in % | 27.0 | 22.8 | 20.3 | 24.2 |
| Macro (>300 mg/g) in % | 6.3 | 4.7 | 5.6 | 8.4 |
| Creatinine in mg/dL | 0.9 (0.8; 1.2) | 1.0 (0.8; 1.3) | 1.0 (0.8; 1.3) | 1.0 (0.8; 1.3) |
| estimated GFR mL/min/1.73m^2^ | 72.5 (53.2; 92.2) | 66.8 (47.7; 84.9) | 67.8 (49.1; 85.2) | 64.7 (46.2; 84.0) |
| <15 | 1.1 | 1.6 | 1.4 | 1.7 |
| 15 to <30 in % | 4.8 | 7.0 | 6.2 | 7.2 |
| 30 to <45 in % | 10.7 | 13.7 | 12.9 | 14.8 |
| 45 to <60 in % | 16.4 | 18.1 | 18.8 | 19.6 |
| 60 to <89 in % | 40.0 | 40.3 | 40.6 | 37.7 |
| ≥90 in % | 27.0 | 19.4 | 20.0 | 19.0 |

Legend: Median (Q1; Q3) or percent (%)

BP = blood pressure; eGFR = estimated glomerular filtration rate; HDL-C = high-density lipoprotein cholesterol; LDL-C = low-density lipoprotein cholesterol; TC = total cholesterol; TG = triglycerides; T2DM = type 2 diabetes mellitus
