# Supplementary material for: Patient and disease characteristics of type-2 diabetes patients with or without chronic kidney disease: an analysis of the German DPV and DIVE databases
Source: Cardiovasc Diabetol. 2019 Mar 16;18:33. doi: 10.1186/s12933-019-0837-x (PMC6420726; doi:10.1186/s12933-019-0837-x)
Supplement: Supplementary file 2 — Additional file 2: Table S2. Drug treatment by comorbidity. Legend: Percent (%). ‡Defined as eGFR < 60 ml/min/1.73 m2 OR eGFR ≥ 60 ml/min/1.73 m2 and overt albuminuria. CKD = chronic kidney disease; DPP-4 = dipeptidyl peptidase-4; GLP-1 RA = glucagon-like peptide-1 receptor agonist; SGLT-2 = sodium-glucose co-transporter-2. [file 12933_2019_837_MOESM2_ESM.docx]

**Table S2:** Drug treatment by comorbidity

|  | Prior stroke | Retinopathy | CAD | CKD^§^ | Diabetic foot complications | PAD |
| --- | --- | --- | --- | --- | --- | --- |
|  | (n=26,270) | (n=18,036) | (n=30,748) | (n=171,930) | (n=38,765) | (n=56,741) |
| Antidiabetic drugs |  |  |  |  |  |  |
| Metformin in % | 30.8 | 32.5 | 32.4 | 28.6 | 32.1 | 31.6 |
| Sulfonylurea in % | 12.2 | 10.4 | 10.4 | 11.2 | 12.0 | 13.7 |
| Alpha-glucosidase inhibitors in % | 1.2 | 1.5 | 1.4 | 1.3 | 1.0 | 1.4 |
| DPP-4 inhibitors in % | 14.1 | 10.9 | 14.8 | 14.5 | 13.9 | 14.3 |
| Glinides in % | 3.9 | 3.3 | 4.0 | 3.9 | 3.6 | 3.8 |
| SGLT-2 inhibitors in % | 1.5 | 2.2 | 2.7 | 2.0 | 3.1 | 2.7 |
| GLP-1 RAs in % | 1.5 | 2.8 | 2.2 | 2.4 | 2.9 | 2.5 |
| Glitazones in % | 0.8 | 1.3 | 1.1 | 1.0 | 0.9 | 1.1 |
| Short-acting insulin in % | 50.8 | 72.0 | 50.9 | 51.4 | 50.8 | 51.2 |
| Long-acting insulin in % | 53.3 | 72.7 | 53.8 | 53.5 | 53.4 | 54.3 |
| ≥2 Antidiabetic drugs in % | 15.6 | 15.0 | 16.4 | 15.0 | 16.7 | 17.6 |
| Antihypertensive drugs in % | 72.5 | 71.0 | 75.4 | 62.6 | 64.0 | 70.0 |
| Angiotensin converting enzyme inhibitors in % | 42.8 | 41.2 | 46.5 | 33.8 | 33.9 | 38.8 |
| Angiotensin receptor blockers in % | 15.4 | 17.6 | 15.6 | 14.2 | 14.6 | 15.7 |
| Beta-blockers in % | 43.3 | 38.6 | 55.9 | 36.7 | 32.7 | 39.3 |
| Calcium channel blockers in % | 24.9 | 23.1 | 20.2 | 19.5 | 19.2 | 21.8 |
| Diuretics in % | 47.2 | 44.9 | 48.3 | 40.2 | 41.0 | 45.7 |
| ≥2 Antihypertensive drugs in % | 56.4 | 53.2 | 63.1 | 47.3 | 45.5 | 52.3 |

Legend: Percent (%)

‡ defined as eGFR <60 ml/min/1.73 m^2^ OR eGFR ≥60 ml/min/1.73m^2^ and overt albuminuria

CKD = chronic kidney disease; DPP-4 = dipeptidyl peptidase-4; GLP-1 RA = glucagon-like peptide-1 receptor agonist; SGLT-2 = sodium-glucose co-transporter-2
